# Adenocarcinoma in a Tailgut Cyst: A Rare Case Report

**DOI:** 10.5146/tjpath.2018.01459

**Published:** 2020-05-15

**Authors:** Songül Şahin, Nuray Kepil, Şebnem Batur, Sibel Erdamar Çetin

**Affiliations:** Department of Pathology, Çankırı Devlet Hastanesi, Çankırı, Turkey; Department of Pathology,Cerrahpaşa University School of Medicine, İstanbul, Turkey

**Keywords:** Tailgut cysts, Developmental cysts, Adenocarcinoma

## Abstract

Developmental cysts are the most common retrorectal area cysts observed in adults. Tailgut cysts tend to be multicystic, and their lining epithelium may display the characteristics of columnar, musin-secreting columnar, ciliated, transitional or squamous epithelia. While the large majority of cysts tend to be benign, several malignant cases have been reported, with adenocarcinoma and carcinoid tumors constituting the more common types of malignant tailgut cysts. A 55-year-old female patient presented to our hospital with complaints of swelling in the gluteal region. Following morphological, histomorphological and immunohistochemical evaluations, a diagnosis of a moderately differentiated adenocarcinoma arising from a tailgut cyst was made. Tailgut cysts are infrequent diseases but adenocarcinoma arising from a tailgut cyst is extremely rare. In rare cases, developmental cysts may undergo malignant transformation that warrants an accurate morphological and histomorphological assessment, as well as numerous samplings, for an accurate diagnosis.

## INTRODUCTION

The retrorectal or presacral area is defined as the region delimited anteriorly by the rectum; posteriorly by the sacrum; superiorly by the peritoneal reflection; inferiorly by the levators ani and coccygeus muscles; and laterally by the ureters and the iliac arteries (1). Masses are rarely found in this area, with a reported incidence of 1 in 40,000 to 63,000 (2). Developmental cysts represent the most common retrorectal area cysts observed in adults, and retrorectal developmental cysts can be classified into three different groups as epidermoid cysts (dermoids), rectal duplication cysts and tailgut cysts (cystic hamartomas) (3).

## CASE REPORT

**Figure 1:**
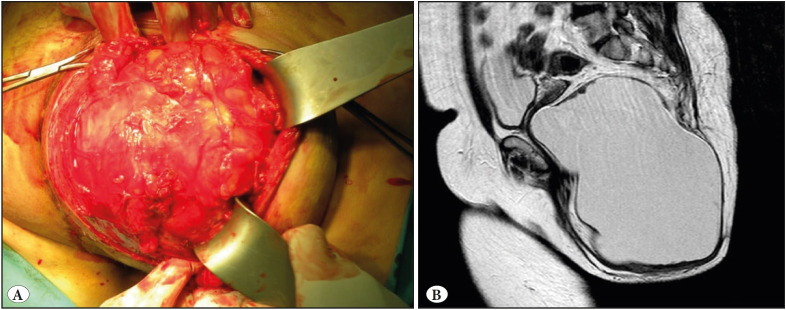
**A)** Macroscopic appearance of the lesion at surgery. **B)** Radiological image of the lesion.

A 55-year-old female patient presented to the General Surgery Outpatient Clinic of the Istanbul University Cerrahpaşa Medical Faculty with complaints of a swelling in the right gluteal area which, while being present since childhood, had gradually started to grow in the past six months. The physical examination of the patient revealed a soft and smooth-surfaced lesion in the right gluteal area that exhibited growth towards the lumen, displacing the vaginal wall on the front side and the anal canal. The patient’s Ca 19-9 value was 204, while other blood chemistry and hemogram results were normal. Magnetic resonance imaging (MRI) revealed a cystic mass lesion 21x16x15 cm in size in the right gluteal region that displaced the anal canal and the distal two-thirds of the vagina, while also deviating the rectum to the left. The lesion had no association with the uterus or adnexa. Medially, the mass reached the external anal sphincter and mesorectum, while reaching the right lateral wall of the vagina and mesorectum superiomedially, and the posterior part of the ischiorectal ramus laterally. The mass was excised as a uniblock through the aspiration of the contained fluids (Figure 1).

A 26x11 cm mass of tissue was clearly observed on macroscopic examination and was surrounded by areas of fat tissue and characterized by a brown-colored membrane with a speckled appearance that had a wall thickness that varied between 0.5 and 1.5 cm. There was no multiocularity in the cyst sections. Nearly 50 percent of the cysts’ inner area was constituted of yellow-orange colored areas of 0.1 to 0.2 cm with irregular boundaries, as well as a rough and granular surface.

**Figure 2:**
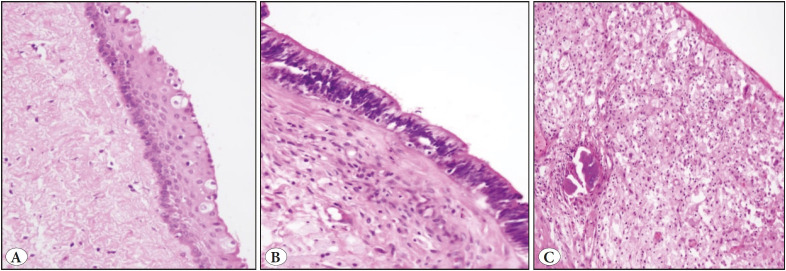
Cyst wall lining consisting of various types of epithelium. **A)** Non-keratinized multi-layered squamous epithelium (H&E; x200). **B)** Columnar epithelium (H&E; x200). **C)** Xanthogranulomatous reaction (H&E; x200).

Histopathological examination revealed the inner surface of the cyst to be lined with non-keratinized multi-layered squamous epithelial, columnar epithelial and transitional epithelial cells, while the wall of the cyst contained connective tissue, vascular structures and slight mononuclear inflammatory cell infiltration (Figure 2).

**Figure 3:**
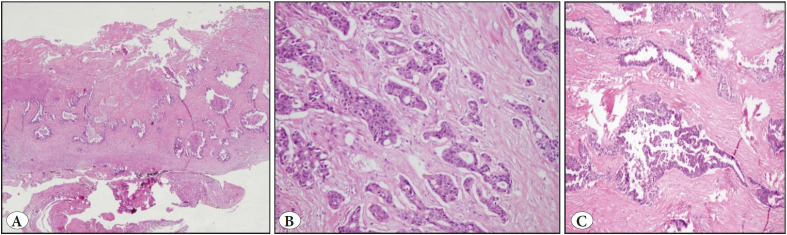
**A)** Tumor invasion over the entire cyst wall (H&E; x40). **B-C)** Moderately differentiated infiltrative-type adenocarcinoma with a tubular pattern (H&E; x100 & x200).

The cyst wall was found to have a moderately differentiated infiltrative-type adenocarcinoma with a tubular pattern, and the tumor showed full-level invasion over the entire cyst wall (Figure 3).

**Figure 4:**
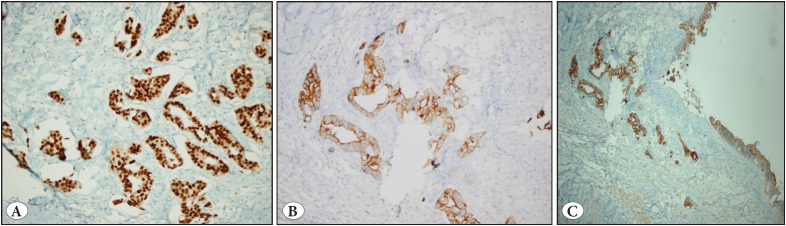
**A)** CDX2 positivity (IHC; x200), **B)** CK7 positivity (IHC; x200), **C)** CK20 positivity (IHC; x200).

The tumor exhibited approximately 10 percent necrosis; stromal reaction was apparent, and the walls adjacent to the tumor and the inner surfaces also exhibited xanthogranulomatous reactions. Immunohistochemical (IHC) tests revealed 100 and 90 percent of the tumor cells to be CDX2 and CK20 positive, respectively, while a moderately high level of tumor cells showed CK7 positivity (Figure 4).

Based on these findings, the case was diagnosed as a moderately differentiated adenocarcinoma arising from a tailgut cyst.

## DISCUSSION

Tailgut cysts normally develop in the eighth week of embryological life from the remains of hindgut involution. While the majority of tailgut cysts occur in the retrorectal area, they may also appear in the anterior rectal, perianal, perirenal and posterior sacral regions.

The lining epithelium of these multicystic lesions may have the characteristics of columnar, musin-secreting columnar, ciliated, transitional and squamous epithelia, with walls of smooth muscle clusters, constituted of fibrous tissue, fat tissue and disorganized bands. No myenteric neural plexus has been identified to date in such cysts. They can be accompanied by granulomatous reactions, which are often associated with an inflammatory infiltrate constituted of mononuclear cells, and more rarely with foreign body-type giant cells (3). 

The majority of cases are in the adult age group, although several cases in childhood or infancy have also been reported. Their distinction from other retrorectal cysts is difficult through imaging methods. In contrast to epidermal and rectal duplication cysts, which tend to be unilocular, tailgut cysts exhibit a multilocular appearance on radiological imaging, and thin calcification has also been observed on their walls in rare instances. A definite diagnosis requires histopathological assessment.

Epidermoid cysts, another type of cyst, have lining epithelia with squamous characteristics. In the presence of skin adnexa on the cyst wall, they are defined as dermoid cysts, and this tends to be their most important histologically distinguishing feature. The cyst walls contain no smooth muscles (1,4,5), and in rectal duplication cysts, the lining epithelial tend to be of the gastric, colonic or respiratory type. No squamous epithelium is observed, and their walls feature organized muscularis propria (6).

While the large majority of developmental cysts tend to be benign, several malignant cases have also been reported. Adenocarcinoma and carcinoid tumors constitute the more common types of malignant tailgut cysts, although cases of neuroendocrine carcinoma, adenosquamous carcinoma, squamous cell carcinoma, endometroid carcinoma and sarcoma have also been reported (4,5,7–9).

Based on the strong p53 and Ki-67 positivity and p21 negativity observed in the dysplastic epithelia of two cases with adenocarcinoma arising from the tailgut, it has been suggested that their sequence of dysplasia and carcinoma follows a similar pattern to that of colon adenocarcinoma (10).

In conclusion, the origin and classification of tailgut cysts continue to be a matter of debate. To date, malignant transformations have been reported in 32 tailgut cysts in the literature, and most of these cases were adenocarcinoma or neuroendocrine tumors, while a few rare cases were carcinoid tumors. Following macroscopic and microscopic examination, we identified a moderately differentiated adenocarcinoma arising from the tailgut, leading us to believe that the accurate identification of areas of malignant changes in these lesions requires a detailed macroscopic examination along with numerous samplings.

## References

[ref-1] Jarboui Slim, Jarraya Hichem, Mihoub Mohamed Ben, Abdesselem Mohamed Morched, Zaouche Abdeljelil (2008). Retrorectal cystic hamartoma associated with malignant disease. Can J Surg.

[ref-2] Fenoglio CM, Noffsinger AE, Stemmermann GN, Lantz PE, Isaacson PG (2008). The Nonneoplastic Anus in Gastrointestinal Pathology: An Atlas and Text.

[ref-3] Killingsworth Christopher, Gadacz Thomas R. (2005). Tailgut cyst (retrorectal cystic hamartoma): report of a case and review of the literature. Am Surg.

[ref-4] Maruyama A., Murabayashi K., Hayashi M., Nakano H., Isaji S., Uehara S., Kusuda T., Miyahara S., Kondo A., Nakano H., Yabana T. (1998). Adenocarcinoma arising in a tailgut cyst: report of a case. Surg Today.

[ref-5] Song Dong Eun, Park Jean Kyung, Hur Bang, Ro Jae Y. (2004). Carcinoid tumor arising in a tailgut cyst of the anorectal junction with distant metastasis: a case report and review of the literature. Arch Pathol Lab Med.

[ref-6] Petras RE (2004). Nonneoplastic intestinal diseases in Sternberg's diagnostic surgical pathology.

[ref-7] Jacob Sunitha, Dewan Yashbir, Joseph Sheela (2004). Presacral carcinoid tumour arising in a tailgut cyst--a case report. Indian J Pathol Microbiol.

[ref-8] Mathieu A., Chamlou R., Le Moine F., Maris C., Stadt J., Salmon I. (2005). Tailgut cyst associated with a carcinoid tumor: case report and review of the literature. Histol Histopathol.

[ref-9] Wöhlke M., Sauer J., Dommisch K., Görling S., Valdix A., Hinze R. (2011). Primary metastatic well-differentiated neuroendocrine tumor arising in a tailgut cyst. Pathologe.

[ref-10] Moreira A. L., Scholes J. V., Boppana S., Melamed J. (2001). p53 Mutation in adenocarcinoma arising in retrorectal cyst hamartoma (tailgut cyst): report of 2 cases--an immunohistochemistry/immunoperoxidase study. Arch Pathol Lab Med.

